# Novel Potential Markers of Metabolic Dysfunction–Associated Steatohepatitis Prone to Hepatocellular Carcinoma

**DOI:** 10.1155/cjgh/7390933

**Published:** 2026-05-26

**Authors:** Chen Ling, Yuya Wang, Susu Liu, Guitao Huo, Yanwei Yang, Nan Xu, Hong Wang, Haoyang Zhao, Zeqiang Zhang, Rui Fu, Yuwei Zhao, Changfa Fan

**Affiliations:** ^1^ Division of Animal Model Research, Institute for Laboratory Animal Resources, National Institutes for Food and Drug Control (NIFDC), Beijing, 102629, China; ^2^ National Center for Safety Evaluation of Drugs, National Institutes for Food and Drug Control (NIFDC), Beijing, 100176, China; ^3^ Division of HIV, AIDS and Sexually Transmitted Virus Vaccines, Institute for Biological Products Control, National Institutes for Food and Drug Control (NIFDC), Beijing, 102629, China; ^4^ Division of Laboratory Animal Monitoring, Institute for Laboratory Animal Resources, National Institutes for Food and Drug Control (NIFDC), Beijing, 102629, China; ^5^ Novogene Co., Ltd., Beijing, 10015, China; ^6^ College of Life Science School, Provincial Key Laboratory of Biotechnology of Shaanxi Province, Northwest University, Xi’an, Shaanxi, 710069, China, nwu.edu.cn

**Keywords:** adverse molecule markers, *Alox5*, *Klk1b4*, malignant, MASH, *Pla2g2e*

## Abstract

**Background:**

Metabolic dysfunction–associated steatohepatitis (MASH), the progressive phenotype of metabolic dysfunction–associated steatotic liver disease (MASLD), is well recognized for its increased risk of progressing to cirrhosis and hepatocellular carcinoma (HCC). However, early diagnosis and identification of the MASH patients with a tendency toward malignancy HCC remain a significant challenge.

**Method:**

In previous studies, we established a novel conditional inducible *HRAS* gene expression murine model with normal diet and lifestyle conditions, which exhibited 100% HCC incidence and recapitulated the four major progression stages of MASH to HCC: MASH, fibrosis, cirrhosis, and HCC. Based on this model, we revealed the potential markers of MASH prone to HCC characteristics of malignant MASH through RNA‐Seq and bioinformatics analysis, and further confirmed by pathological biopsy, biochemical tests, inflammatory cytokines measurement, Oil O red staining, and immunohistochemical examination.

**Results:**

For MASH, the initial stage of HCC, we observed evidence of hyperlipidemia and insulin resistance in the HRAS mouse model based on blood morphology, biochemical parameters, and insulin tolerance test. Furthermore, the pathological features of MASH were confirmed by the presence of hepatocellular fatty vacuolar changes, lipid droplet accumulation, and inflammation. Then, RNA‐Seq analysis revealed the molecular signatures of malignant MASH and revealed three novel potential markers associated with adverse progression of MASH to HCC: *Klk1b4, Alox5*, and *Pla2g2e*.

**Conclusion:**

Based on a stable and novel murine model of MASH prone to HCC, we, for the first time, presented a comprehensive molecular signature and identified novel potential adverse progression markers of MASH prone to malignant HCC, which contributed possibly to early clinical diagnosis and prognostic assessment, even becoming potential therapeutic target.

## 1. Introduction

Metabolic dysfunction–associated steatotic liver disease (MASLD) has become the most prevalent liver disease worldwide, with an estimated global prevalence of 38% among adults and approximately 13% in children and adolescents [1, 2]. MASLD is an umbrella term starting from simple steatosis and frequently progresses to metabolic dysfunction–associated steatohepatitis (MASH) characterized by persistent liver injury, inflammation, and fibrosis, which may progress to cirrhosis and hepatocellular carcinoma (HCC) latterly [3, 4]. MASH has been increased by up to 4‐fold over the past decade, compared with a 2.5‐fold increase for viral hepatitis [5]. Specifically, the Middle East was found to have the greatest frequency of MASH (31.79%), followed by Asia (34%), South‐East Asia (33%), the Pacific region (28%), and East Asia (30%) [6].

For MASH patients, few of them have specific symptoms, early diagnosis of patients with a tendency toward malignant HCC holds significant clinical value, but it presents clear technical challenges [7]. The majority of MASH was considered to have a benign outcome and approximately 40% progressed to fibrosis or HCC, resulting from “multiple hits”, such as lipotoxicity, oxidative stress, and inflammation [8–11]. The American Association for the Study of Liver Diseases (AASLD) emphasizes that evaluation of hepatic fibrosis stage remains crucial for predicting MASH‐related events and evaluating disease severity [12].

The Asian Pacific Association for the Study of the Liver (APASL) pointed out that “At Risk MASH” (MASH and significant fibrosis, F2/F3 stage) was urgently needed in clinical trials [13]. Currently, noninvasive tests (NITs) were used to identify patients with advanced liver fibrosis (F3‐4) who are present in 2%–5% of the primary care population [14]. However, the uncleared standardized cutoff for diagnostic accuracy for advanced fibrosis, coupled with the lack of highly sensitive NITs, presents significant challenges in the identification of advanced fibrosis [13]. Therefore, the illustration of malignant MASH characteristics, molecular signatures in early stages, and adverse predictable progression markers play important roles.

RAS protein has functions in converting GTP to GDP to transduce signal to regulate cell growth [15]. Activated RAS proteins are capable of interacting with multiple proteins in the downstream effector family [16], thereby modulating cellular metabolism via the PI3K/AKT and RAF/MEK/ERK signaling pathways, including glycolytic metabolism, the tricarboxylic acid cycle, and lipid metabolism. *HRAS* gene was considered as a novel key driver in the insulin‐like growth factors (IGFs)/insulin resistance (IR) axis‐associated subnetworks [17]. Overexpression of the wild‐type *HRAS* gene may serve as a prominent causative factor for MASH‐related HCC, despite *HRAS* gene mutations being considered the primary mechanism of carcinogenesis [18].

Previously, we had established an HCC mouse model based on a novel conditional tamoxifen (TAM) inducible *HRAS* gene overexpression system under normal diet and lifestyle [19]. This mouse model demonstrates the full sequential progressions from MASH to HCC rapidly after the initiation of *HRAS* expression, which might be a useful and reliable tool for the identification of MASH prone to worse progression. Specifically, it represented hyperlipidemia, IR, and vacuolar degeneration in week 1 after *HRAS* expression; liver fibrosis and inflammation in week 2–3; and HCC in week 4, approximately. In this work, we attempted to reveal the potential adverse progression markers of malignant MASH, aimed to give new clues for the early diagnosis and prognostic evaluation.

## 2. Results

### 2.1. The General Characteristics Align With Clinical Manifestation of MASH

In our early studies [19], a mouse model (named HRAS‐HCC mouse) was established by insertion of wild‐type human *HRAS* gene into C57BL/6 strain genome, via crossing with Cre‐ERT2 mice, resulting in the specific overexpression of *HRAS* in liver after TAM induction (Figure 1(a)). The 5‐week‐old mice were randomized into the following two groups: *HRAS* overexpression induced by the TAM(HRAS^TAM^) group as experiment for malignant MASH and corn oil (HRAS^non−TAM^) group as control (Figure 1(b)). The expression of HRAS was confirmed to be significantly elevated compared to the HRAS^non−TAM^ group (*p* < 0.05) (Figures 1(c), 1(d), 1(e)). Hepatomegaly, perivascular hepatic steatosis characterized by vacuolar changes in hepatocytes, and well‐defined HCC were observed in liver tissues of HRAS^TAM^ mice (Figure 1(f)). Liver weight and liver/body weight ratio were increased (*p* < 0.0001, Figures 1(g) and 1(h)). However, no statistically significant differences in survival time or body weight were represented at this stage (Figures 1(i) and 1(j)), which is consistent with the clinical observation that no apparent symptoms occur during the early stage of HCC [9, 20]. Taken together, the phenotype HRAS‐HCC mouse was like that of clinical MASH patients, indicating its usefulness and reliability for discovery of molecular markers of MASH prone to worse progression.

FIGURE 1Basic manifestations of MASH in HRAS mouse model. (a) Schematic graph illustrating the detailed information of the inducible HRAS system. (b) Sketch map of group information. The intraperitoneal injection of tamoxifen‐induced HRAS overexpression in mice, named as HRAS^TAM^ group; corn oil as solvent, named as HRAS^non−TAM^ group. (c) The expression of HRAS gene in liver tissue on mRNA level by RT‐qPCR (*n* = 3, *p* < 0.05). (d) The expression of HRAS gene in liver tissue on protein level by WB (*n* = 3). (e) The gray value analysis of WB bands (*n* = 3, *p* < 0.001). (f) The fresh liver tissues and H&E staining of HRAS mice model in the stage of MASH and HCC. The white dashed line represents the HCC outline (*n* = 3). (g) Liver weight of HRAS^TAM^ (*n* = 9) and HRAS^non−TAM^(*n* = 8). (h) Liver/body weight ratio (*p* < 0.0001) of HRAS^TAM^ (*n* = 9) and HRAS^non−TAM^(*n* = 8). (i) Survival of HRAS^TAM^ (*n* = 34) and HRAS^non−TAM^(*n* = 21). (j) Body weight of HRAS^TAM^ (*n* = 37) and HRAS^non−TAM^(*n* = 21) in Days 1–7. ^∗^ *p* < 0.05; ^∗∗^ *p* < 0.01; ^∗∗∗^ *p* < 0.001; ^∗∗∗∗^ *p* < 0.0001; ns: no statistical difference.

**Figure:**
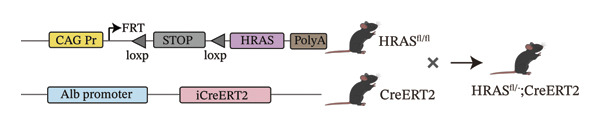
(a)

**Figure:**
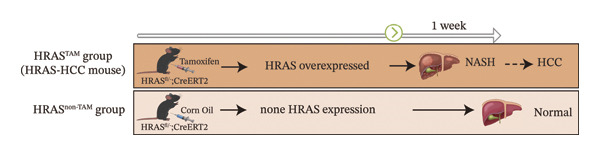
(b)

**Figure:**
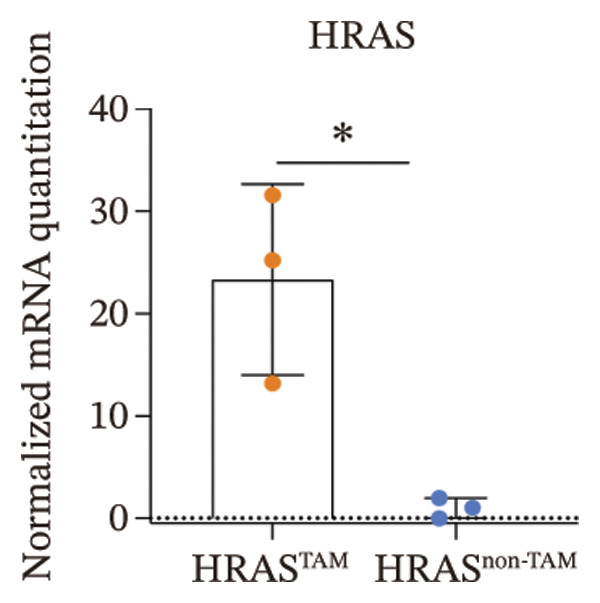
(c)

**Figure:**
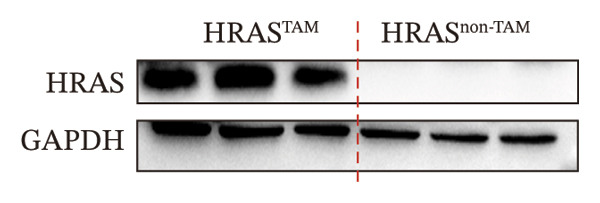
(d)

**Figure:**
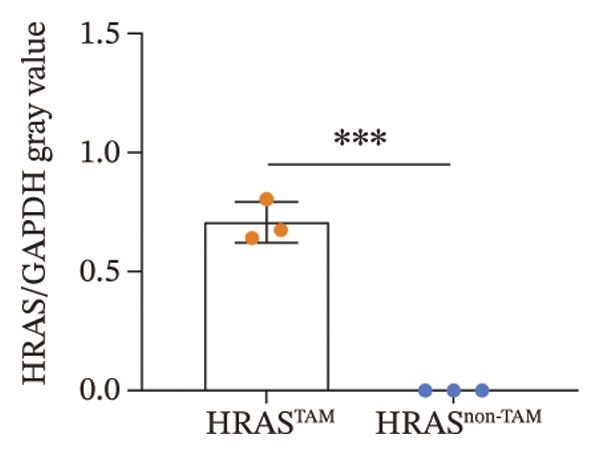
(e)

**Figure:**
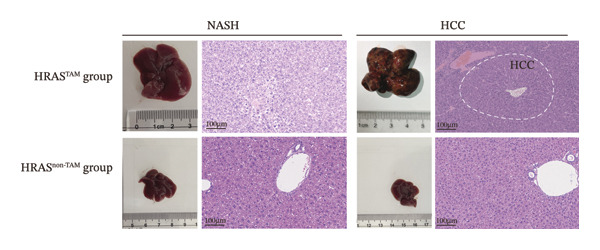
(f)

**Figure:**
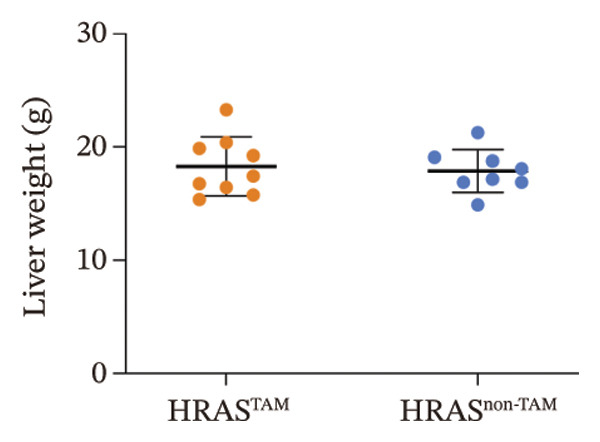
(g)

**Figure:**
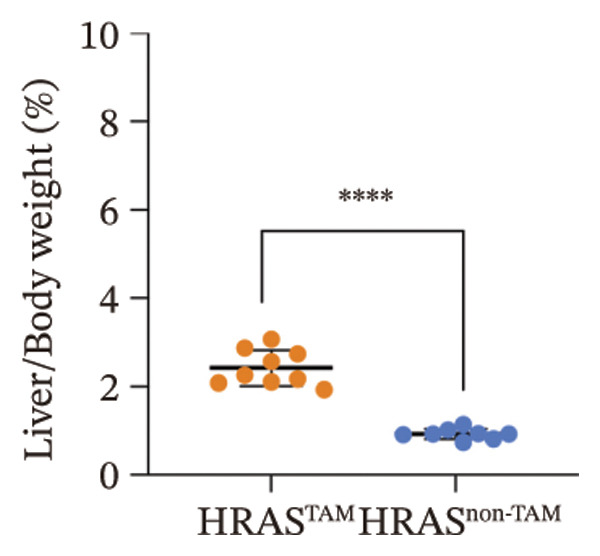
(h)

**Figure:**
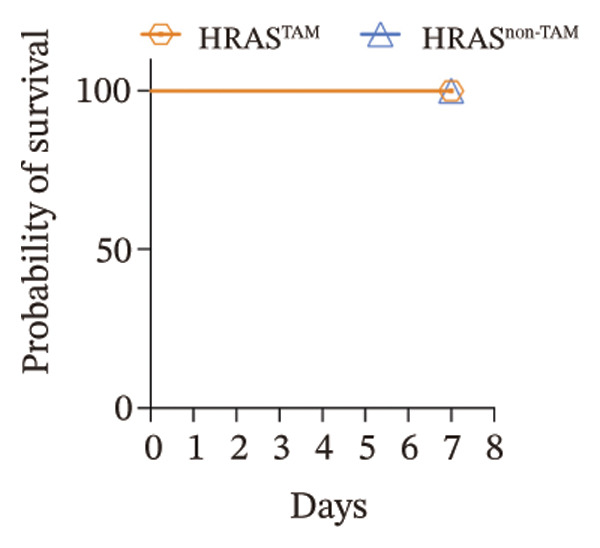
(i)

**Figure:**
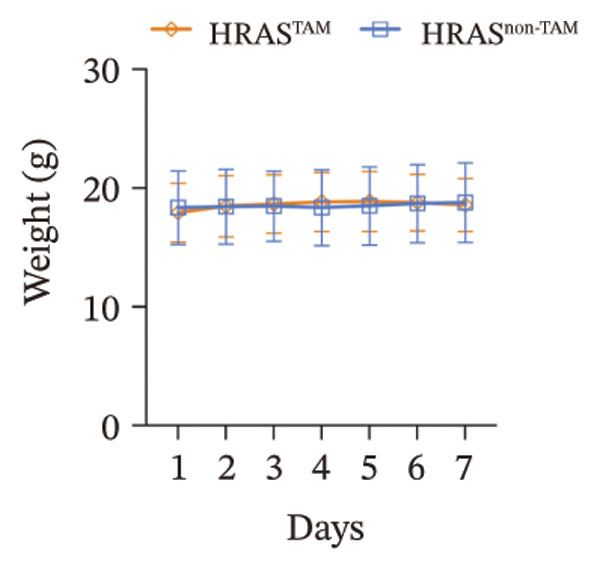
(j)

### 2.2. The Physiology and Biochemical Markers Favor the Features of MASH

As is well recognized, MASH patients exhibit hyperlipidemia, abnormalities in biochemical indices and markers, as well as dysregulation of inflammatory cytokines [20]. To confirm the consistency with the clinical patients, we further investigated the physiological and biochemical features of the HRAS‐HCC mouse model. Chylemia were observed in HRAS^TAM^ mice (Figure 2(a)), while the serum cholesterol (CHO) and triglyceride (TG) were significantly elevated (*p* < 0.0001 and *p* < 0.001, Figures 2(b) and 2(c)). However, no significant alterations of high‐density lipoprotein cholesterol (HDL‐C) or low‐density lipoprotein cholesterol (LDL‐C) were observed in HRAS^TAM^ mice (*p* = 0.3909 and *p* = 0.5817, Supporting Figure 1A‐1B). Furthermore, MASH marker gene *Apoa5* (*p* < 0.05), *Cyp7a1* (*p* < 0.05), and *Lpl* (*p* = 0.4835) were downregulated in HRAS^TAM^ mice (Figures 2(d) and 2(e), Supporting Figure 1C), consistent with previous studies [21, 22].

FIGURE 2The physiology and biochemical changes advanced the features of MASH. (a) The serum appearance of HRAS^TAM^ (*n* = 4) and HRAS^non−TAM^ (*n* = 4). (b‐c) The serum biochemical indexes of HRAS^TAM^ (*n* = 6) and HRAS^non−TAM^ (*n* = 8). CHO (*p* < 0.0001) in (b); TG (*p* < 0.001) in (c). (d) mRNA expression of *Apoa5* (*p* < 0.05) in HRAS^TAM^ (*n* = 4) and HRAS^non−TAM^ mice (*n* = 4). (e) mRNA expression of *Cyp7a1* (*p* < 0.05) in HRAS^TAM^ (*n* = 4) and HRAS^non−TAM^ mice (*n* = 4). (f) The inflammatory cytokines in HRAS^TAM^ (*n* = 6) and HRAS^non−TAM^(*n* = 4). Ccl11 (*p* < 0.05) in (f); Cxcl1 (*p* < 0.05) in (g); IL10 (*p* < 0.01) in (h); and IL2 (*p* < 0.01) in (i). (j) Scheme of insulin tolerance test and the change of blood glucose in HRAS^TAM^ (*n* = 6) and wild‐type mice (*n* = 5). ^∗^ *p* < 0.05; ^∗∗^ *p* < 0.01; ^∗∗∗^ *p* < 0.001; ^∗∗∗∗^ *p* < 0.0001; ns: no statistical difference.

**Figure:**
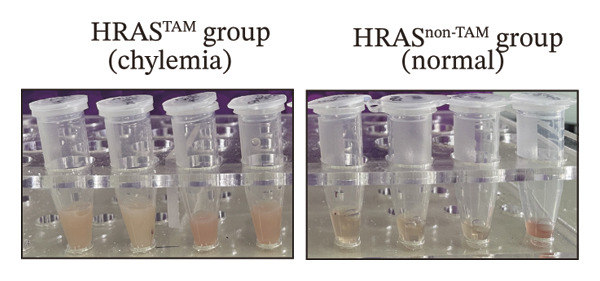
(a)

**Figure:**
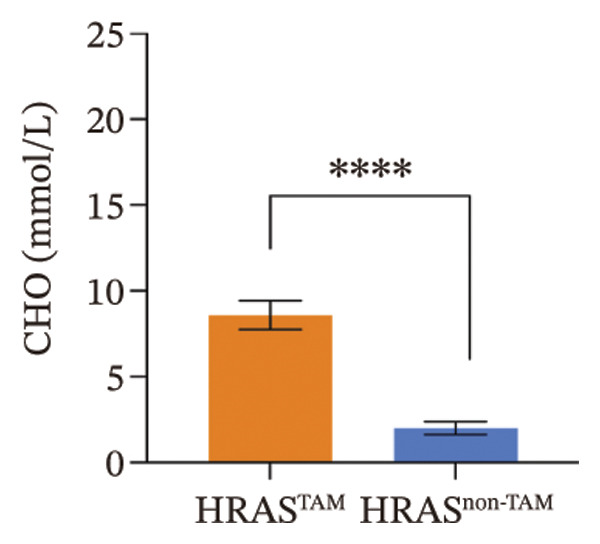
(b)

**Figure:**
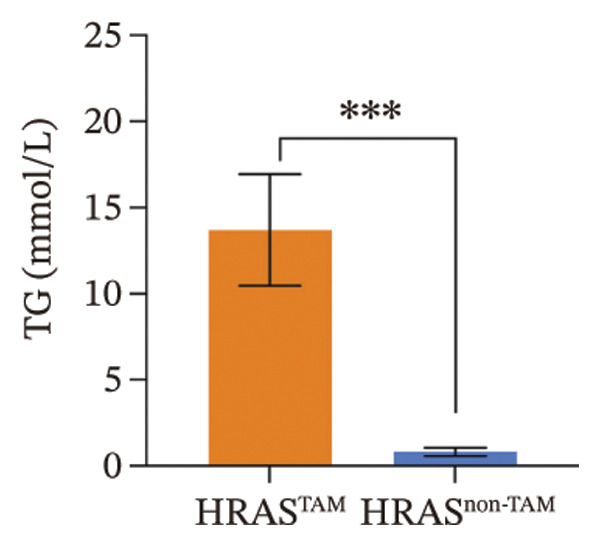
(c)

**Figure:**
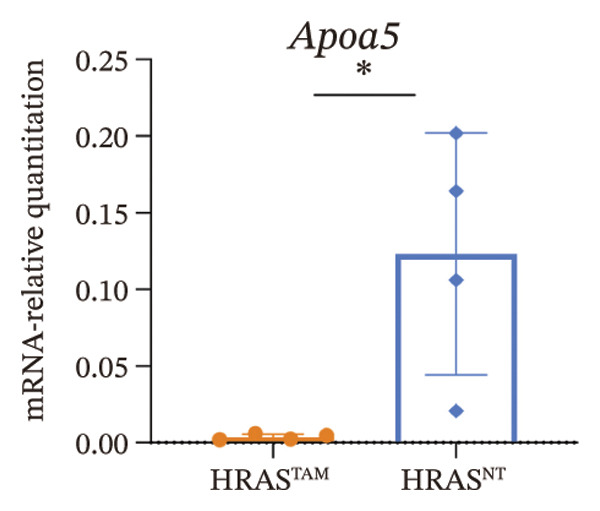
(d)

**Figure:**
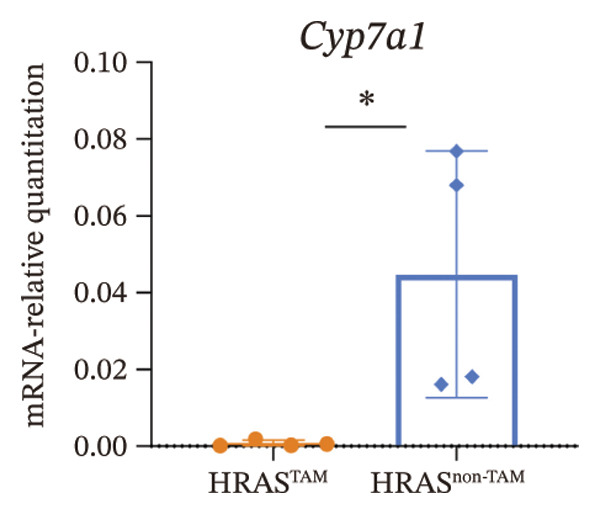
(e)

**Figure:**
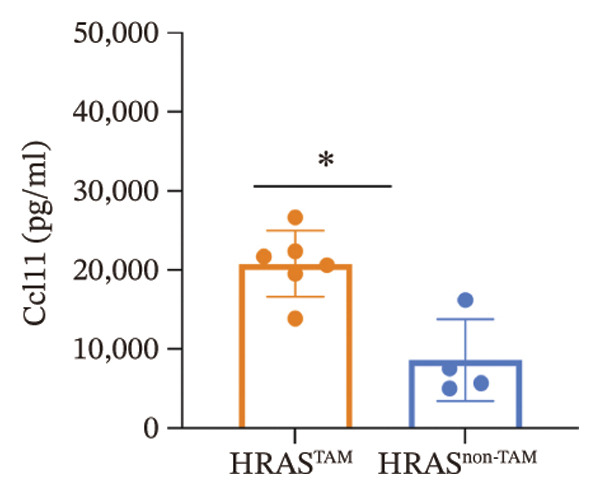
(f)

**Figure:**
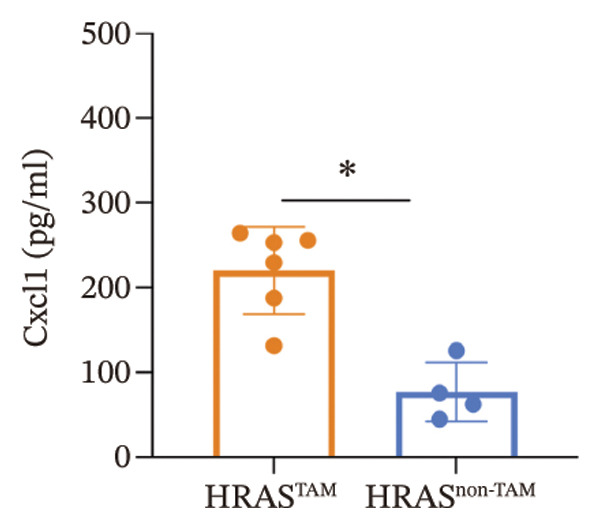
(g)

**Figure:**
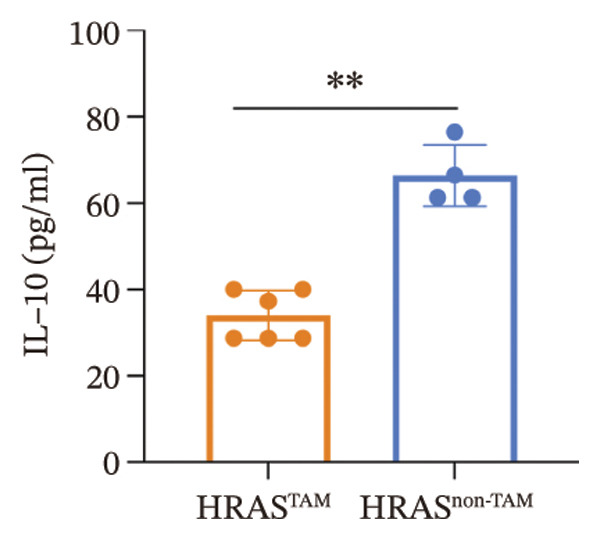
(h)

**Figure:**
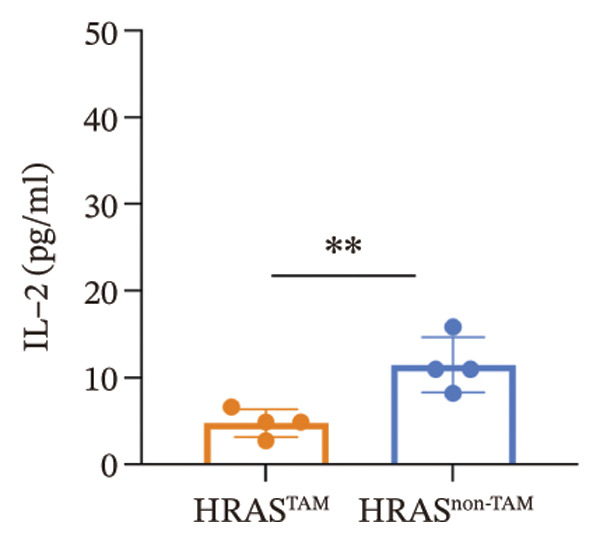
(i)

**Figure:**
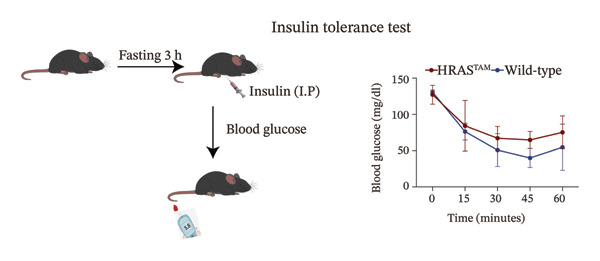
(j)

Elevated secretion of inflammatory factors by adipocytes and macrophages is one of the key pathological features of MASH [23]. Pro‐inflammatory cytokines Cxcl1 (*p* < 0.05), Ccl11 (*p* < 0.05), IL6 (*p* = 0.2188), and Ccl2 (*p* = 0.6044) were upregulated in HRAS^TAM^ mice (Figures 2(f) and 2(g), Supporting Figure 1D‐1E). Meanwhile, anti‐inflammatory cytokines IL2 (*p* < 0.01), IL10 (*p* < 0.01), IL3 (*p* = 0.2757), IL13 (*p* = 0.0837), IL12p70 (*p* = 0.3352), and Csf2 (*p* = 0.3741) were downregulated in HRAS^TAM^ mice (Figures 2(h) and 2(i), Supporting Figure 1F–1I).

To confirm whether insulin tolerance exists in this mouse model, which is common in MASH patients, insulin tolerance test (ITT) was performed after fasting 3 h (Figure 2(j)). In fasting state, HRAS^TAM^ mice (*n* = 6) exhibited a slower rate of blood glucose decline after administration of exogenous insulin (0.75 U/kg, intraperitoneal), with a reduction of approximately 30% compared to the initial blood glucose level and a relatively small variation (Figure 2(j)), which is consistent with the previous results [24]. In contrast, wild‐type mice (*n* = 5) presented a faster rate of blood glucose reduction, with a decrease of approximately 40% compared to the initial blood glucose level and a relatively large variation (Figure 2(j)). It suggested that insufficient insulin secretion may occur in HRAS^TAM^ mice. In summary, these results of physiology and biochemical markers favored the features of MASH.

### 2.3. Histopathological Manifestations Correlate With MASH

Pathological features are an important parameter for clinical diagnosis of MASH, which is determined by hepatocellular steatosis, including macrovesicular and microvesicular steatosis [25]. Compared with the HRAS^non−TAM^ group, the liver tissue of HRAS^TAM^ mice exhibited hepatomegaly and presented a greasy surface (Figure 3(a)). Histological examination via H&E staining revealed extensive microvesicular steatosis in the liver tissues of HRAS^TAM^ mice (Figure 3(b)). Lipid droplet deposition was observed peripherally around hepatic arteries and veins (Figures 3(c) and 3(d)). The stronger intensity of LPL occurred in the liver tissue of HRAS^TAM^ mice (Figure 3(e)). Collectively, the data indicated that pathological manifestations of this murine model coincided with MASH.

FIGURE 3Pathological characteristics consistent with MASH. (a) Morphology of the fresh liver in HRAS^TAM^ and HRAS^non−TAM^ mice at week 1. (b) Pathological analysis of MASH characteristics in the liver of HRAS^TAM^ and HRAS^non−TAM^ mice by H&E staining at Week 1. The magnified area is shown in black box in the bottom left corner. Black asterisks marked the magnified area, respectively (*n* = 3). Scale bar: 100 μm. (c) Lipid droplets by oil red O staining in the liver tissue of HRAS^TAM^ and HRAS^non−TAM^ mice (*n* = 2). Scale bar: 100 μm, 50 μm, and 10 μm. (d) Lipid droplets by BODIPY staining in the liver tissue of HRAS^TAM^ and HRAS^non−TAM^ mice (*n* = 2). Scale bar: 100 μm, 50 μm, and 10 μm. (e) The expression of LPL on protein level by IHC in the liver tissue of HRAS^TAM^ and HRAS^non−TAM^ mice (*n* = 2). Scale bar: 250 μm, 100 μm, and 50 μm.

**Figure:**
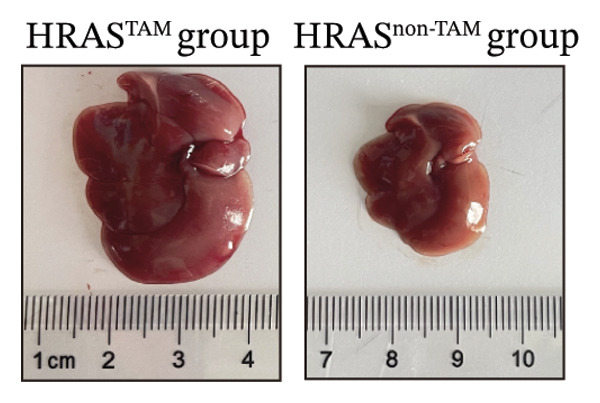
(a)

**Figure:**
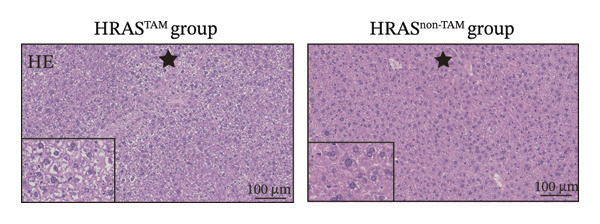
(b)

**Figure:**
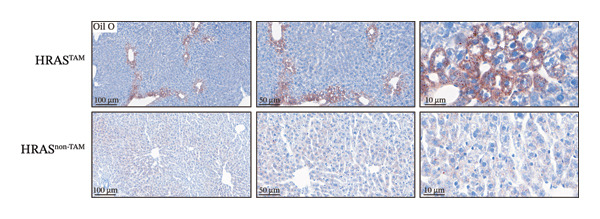
(c)

**Figure:**
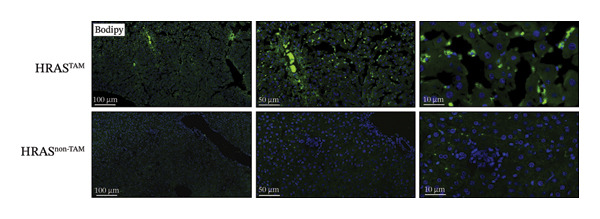
(d)

**Figure:**
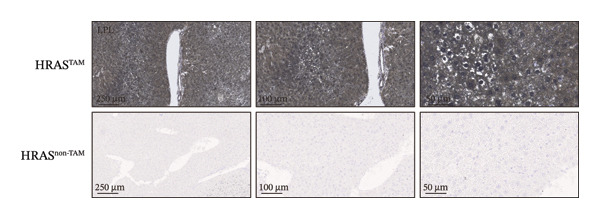
(e)

### 2.4. The Holistic Molecular Signatures of MASH Prone to Malignancy

To further elucidate the holistic molecular features of MASH, the transcriptome profiling of the liver tissues was conducted (Figure 4, Supporting Table 2). The genes of HRAS^TAM^ and HRAS^non−TAM^ groups were divided into two different clusters by PCA (Figure 4(a)), indicating that the expression profile of genes undergoes significant changes. A total of 1691 differentially expressed genes (DEGs), including 943 upregulated and 748 downregulated, were identified under the criteria of adjusted *p* < 0.05 and a |log_2_ fold‐change FC| ≥ 1.5 (Figure 4(b), Supporting Table 3). Subsequently, these DEGs were subjected to Kyoto Encyclopedia of Genes and Genomes (KEGG) and Gene Ontology (GO) functional enrichment analysis (Figures 4(c) and 4(d), Supporting Table 4). The MASH‐related pathways, including retinol metabolism, PPAR signaling, arachidonic acid (AA) metabolism pathway, and inflammatory mediator regulation of transient receptor potential (TRP) channels (Figure 4(c)), fatty acid metabolic and unsaturated fatty acid metabolic (Figure 4(d)) were found to be enriched, which are consistent with those of previous studies [26–29]. Furthermore, 103 DEGs were enriched in the MASH‐related pathways of KEGG, as well as 196 DEGs were enriched in GO analysis. Thus, the 65 common DEGs enriched in both KEGG and GO were overlapped and showed in heat map, including 18 upregulated and 47 downregulated genes (Figures 4(e) and 4(f), Supporting Table 4). Previous studies demonstrated that the upregulation of *Acsl4* contributes to hepatic stellate cell (HSC) activation by altering cellular lipid composition, thereby promoting the development of MASH‐HCC [30]. Notably, the expression of *Acsl4* also elevated in HRAS‐HCC mice at the MASH stage (Figure 4(e), Supporting Table 4), which is consistent with previous reports [30]. Moreover, the top 5 significant downregulated genes *Cyp4a10*, *Rdh16*, *Acot1*, *Acot3*, and *Srd5a1* have been reported to be associated with MASH, abnormal lipid metabolism, or liver injury [31–35]. Those results indicated the consistency and reliability of this study in relation to clinical observations and previous research. The other genes in Figure 4(e), *Nat8*, *Ggt6*, *Cyp2c50*, *Cyp2j5*, *Alox5*, *Pla2g2e*, *Akr1c18*, *Sult1e1, Rdh11, Klk1b4*, *Srd5a2, Gstp2, Cyp2a4*, *Chac1*, *Cyp2c37*, *Cyp2c23*, and *Cyp2d11* were proposed firstly in our study, prompting that they might be the potential adverse progression markers.

FIGURE 4The molecular signatures by transcriptomics functional analysis of the malignant transformed MASH tendency. (a) Principal component analysis of RNA‐Seq data from the liver tissue of HRAS^TAM^ (*n* = 5) and HRAS^non−TAM^ mice (*n* = 4) divided into different clusters. (b) Volcano plots of DEGs in the liver of HRAS^TAM^/HRAS^non−TAM^ mice at Week 1 (adjusted *p* < 0.05, Log_2_|fold‐change| > 1.5). (c) KEGG pathway enrichment analysis of DEGs in the liver of HRAS^TAM^/HRAS^non−TAM^ mice at Week 1. (d) The gene count of MASH‐related top 10 biological process (BP) in GO enrichment analysis. (e) Venn diagram and heat map of the 65 intersection DEGs information between GO and KEGGE analysis. (f) Gene differential expression sorting chart of the 65 intersection genes of GO and KEGGE analysis in (e). The order was sorted according to the amount of expression (Log_2_|fold‐change|). Top 5 increased genes in black box were marked by red, and Top 5 decreased genes were marked by blue.

**Figure:**
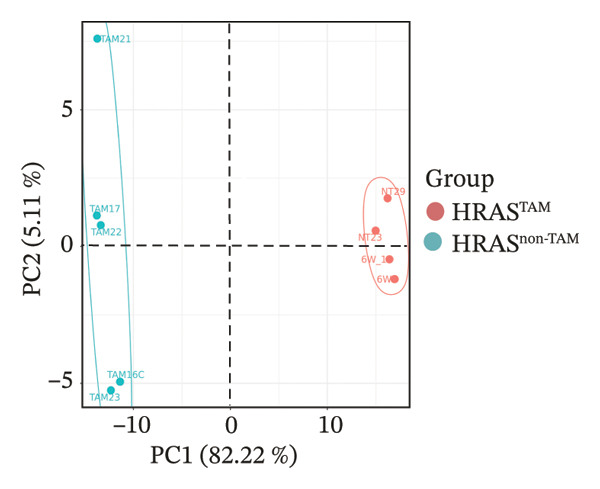
(a)

**Figure:**
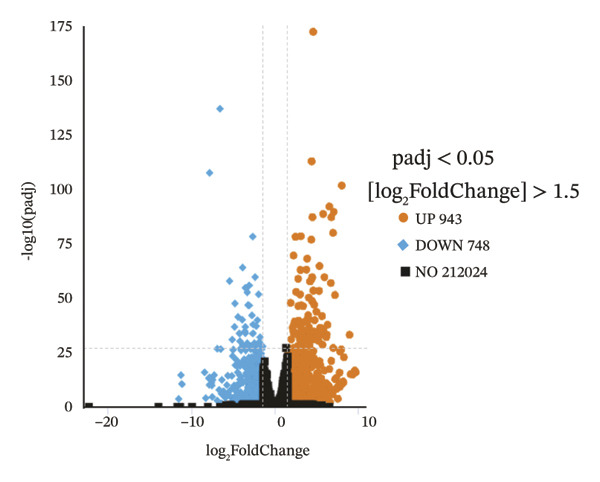
(b)

**Figure:**
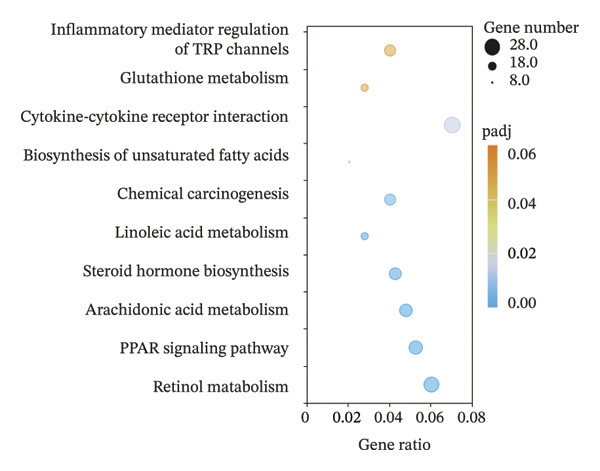
(c)

**Figure:**
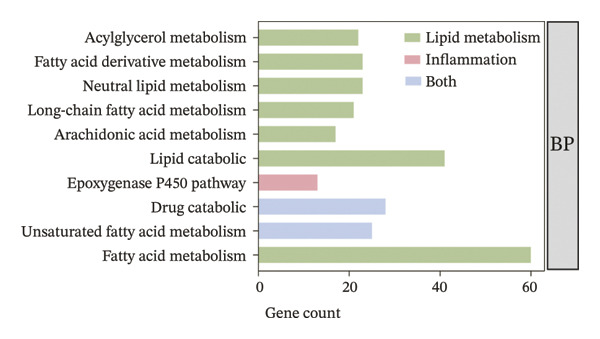
(d)

**Figure:**
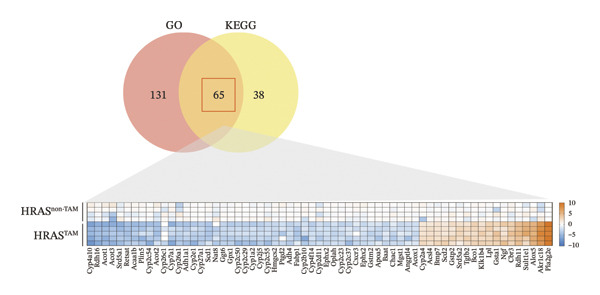
(e)

**Figure:**
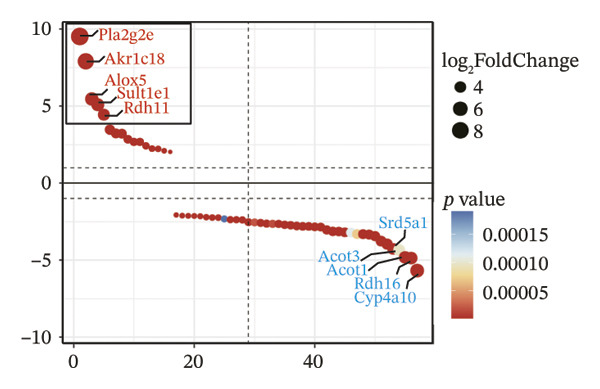
(f)

### 2.5. Novel Potential Markers of MASH Prone to HCC Malignancy

To identify predictable markers of the progression of MASH, the represented top 10 upregulated genes identified through RNA‐Seq, *Alox5*, *Pla2g2e*, *Akr1c18*, *Sult1e1, Rdh11, Cbr3, Ngf, Gsta1, Lpl*, and *Klk1b4*, were selected for RT‐qPCR validation (Figures 5(a), 5(b), 5(c), 5(d), 5(e) and Supporting Figure 2). The expressions pattern of seven genes, *Alox5*, *Pla2g2e*, *Akr1c18*, *Sult1e1, Rdh11, Lpl*, and *Klk1b4*, was identical with the sequencing data. MASH is a well‐known disease that goes through “multiple hits” such as lipotoxicity, oxidative stress, and inflammation [8–11]. Since the cytokine secretion plays a crucial role in the progression of MASH, we further wanted to know whether the seven genes involved in the process of inflammatory response by PPI network analysis. The *Lpl, Klk1b4, Alox5*, and *Pla2g2e* genes were found to participate in the release of cytokines IL2/3/6/10/13, Ccl2/11, and Csf2 (Figure 5(f)). Upregulation of *LPL* has been extensively documented to contribute to the malignant progression of MASH through lipotoxicity and IR, which are mediated by the production of IL6 and Ccl2 [36]. Notably, the concentration of IL6, Ccl2, and Ccl11 was detected higher in HRAS^TAM^ mice than that in HRAS^non−TAM^ mice, suggesting that the novel genes *Klk1b4, Alox5*, and *Pla2g2e* might enhance the production of IL6, Ccl2, and Ccl11, thereby contributing to adverse progression of MASH. To sum up, the expressions of *Klk1b4, Alox5*, and *Pla2g2e* genes might serve as potential indicators for the adverse progression of MASH.

FIGURE 5Validation of novel potential markers of MASH prone to malignancy. (a–e) Verification of the top 10 increased intersection genes by RT‐qPCR. *Pla2g2e* in (a), *Akr1c18* in (b), *Alox5* in (c), *Sult1e1* in (d), and *Rdh11* in (e) (*n* = 4). ^∗^ *p* < 0.05; ^∗∗^ *p* < 0.01; ^∗∗∗^ *p* < 0.001; ^∗∗∗∗^ *p* < 0.0001; ns: no statistical difference. (f) The PPI network by STRING web tool mapped the relationships between the top 5 increased and decreased intersection genes with inflammatory cytokines. The cycle indicated the enriched pathways. (g) Schematic diagram of the potential mechanism in HRAS‐HCC model. Overexpressed HRAS was marked by yellow. The MASH progression‐related biological processes that have been reported in the literature were marked by green. The potential marker genes and interactions in this manuscript were marked by pink and dashed black lines, respectively.

**Figure:**
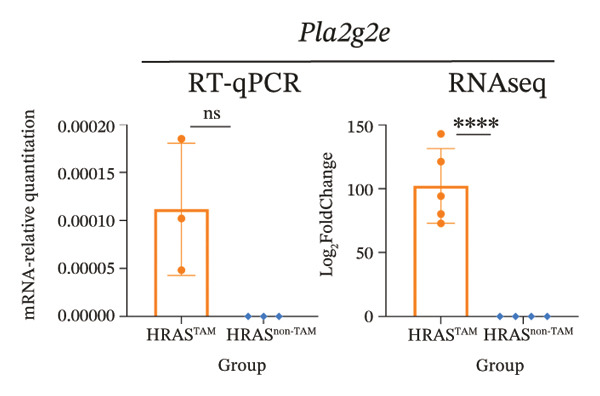
(a)

**Figure:**
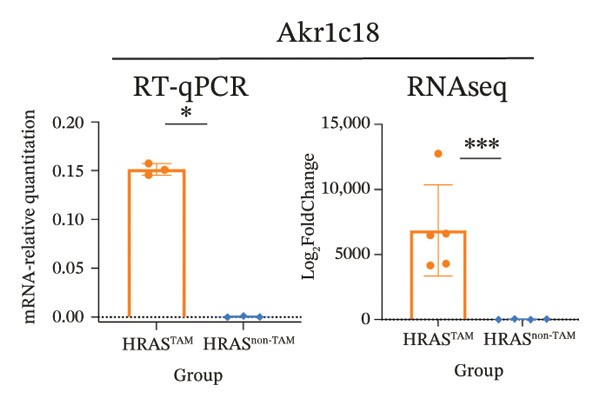
(b)

**Figure:**
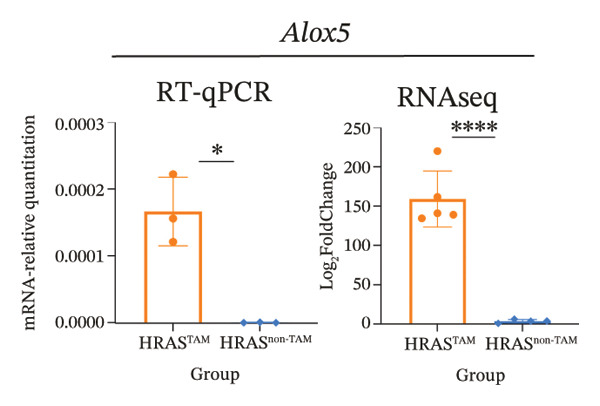
(c)

**Figure:**
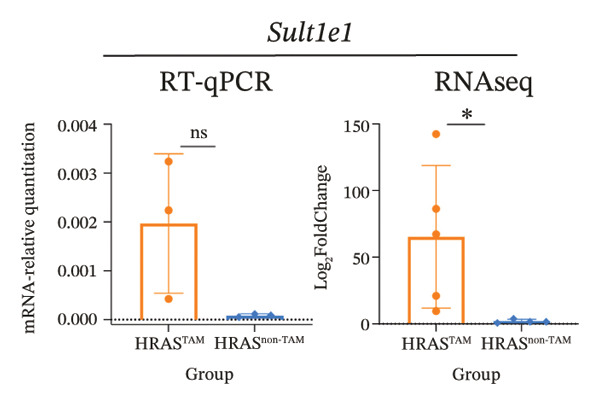
(d)

**Figure:**
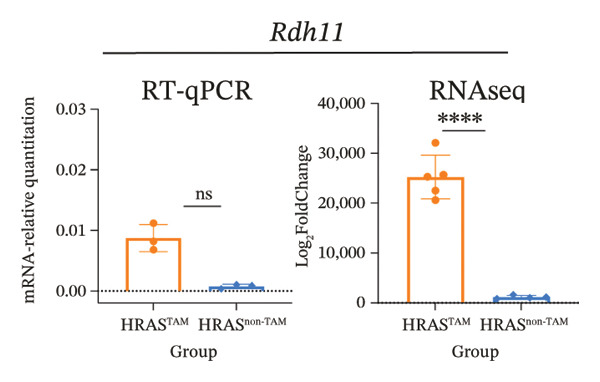
(e)

**Figure:**
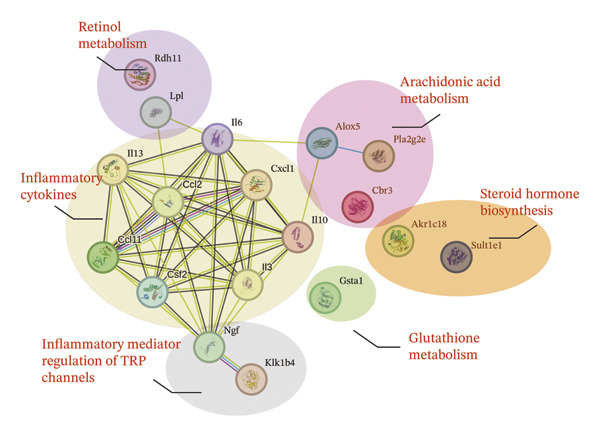
(f)

**Figure:**
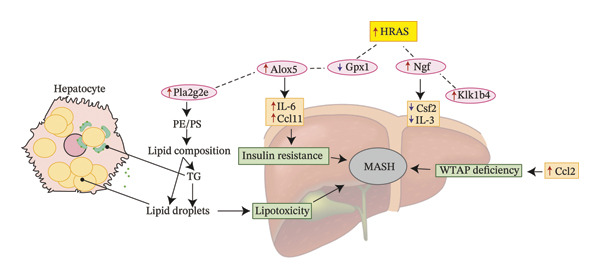
(g)

To further investigate the association between the three potential marker genes *Klk1b4, Alox5*, and *Pla2g2e* genes, with HRAS gene, the interaction between *HRAS* and the 65 DEGs was further analyzed by PPI network (Supporting Figure 2D). The top 10 up/downregulated genes were marked by red/blue cycle, respectively. Ngf‐Klk1b4 and HRAS‐Gpx1‐Alox5‐Pla2g2e axes may potentially be involved in the promotion of MASH development by HRAS overexpression (Figure 5(g)). To extend the translational relevance of these findings to human disease, we subsequently interrogated public databases to evaluate the expression and prognostic significance of these genes in patient cohorts. The high expression of *ALOX5* and *PLA2G2E* is significantly associated with poor prognosis in patients with nonalcoholic, non‐hepatitis‐related HCC (Supporting Figure 2E‐2F). These genes may be coregulated with or regulated by HRAS and may participate in the biological processes associated with MASH prone to malignancy.

## 3. Discussion

MASH has progressively emerged as a significant risk factor for liver carcinogenesis [37]. Currently, limited studies elucidated the underlying key molecular events upon the malignant transformation trend of MASH into HCC [38]. In humans, Ras proteins participate in various physiological processes related to cell growth, division, and survival [39]. The RAS/ERK signaling pathway was a critical signaling cascade in adipogenesis [40]. The overexpression of HRAS in cells can upregulate the activity of N‐acetylglucosaminyltransferase‐V (GnT‐V), leading to enhanced cell proliferation, reduced adhesion to fibronectin, increased adhesion to laminin, and elevated invasiveness through Matrigel [41]. Furthermore, CREBH glycosylation mediated by GnT‐V expression could significantly affect the expression of PPARα and SCD1 involved in lipogenesis, lipotoxicity, and inflammation, thereby exacerbating liver damage by inhibiting glycosylation in both cultured cells and mouse models of MAFLD [42]. Meanwhile, activating mutations in HRAS (Q61R and Q61K) promotes hepatocyte proliferation and the development of MAFLD‐HCC by activating RAS/MAPK and PI3K/PDK1/AKT pathways, as demonstrated in the MAFLD mouse model [43]. Therefore, our study utilized a mouse model of MASH tends to HCC with 100% incidence to systematically characterize the molecular signature and identify novel potential biomarkers of MASH prone to malignancy firstly.

Early diagnosis of MASH remains extremely challenging [44, 45]. In clinic, patients exhibiting hepatomegaly, hyperlipidemia, hypertriglyceridemia, hepatocyte vacuolar transformation, and inflammation were diagnosed with MASH [46]. In this work, the HRAS^TAM^ mice displayed hyperlipidemia, elevated levels of CHO and TG, as well as IR (Figures 2(a), 2(b), 2(c)), indicating that the murine model could effectively simulate the characteristics of clinical MASH patients and was a good model tool. Currently, high‐risk genetic factors *Pnpla3*, *Lpl*, *Sod2*, *Apoa5*, *Acly*, *Lipin*, *Scd1*, *Nr1h3*, and *Cyp7a1* have been reported [47]; however, the underlying mechanisms and significance upon MASH to HCC still need to be further elucidated. We described the molecular signatures of malignant MASH from a holistic view (Figures 4 and 5). The 1691 DEGs were mainly enriched in 14 lipid metabolism‐ and 5 inflammation‐related pathways and biological processes via KEGG and GO analyses (Figures 4(c) and 4(d)). For example, retinol, also known as vitamin A, has been gradually considered as key regulators of glucose and lipid metabolism in the liver and adipose tissue [48, 49]. An altered retinol metabolism has been identified as one of the different pathways involved in the complex process of MASH‐developed hepatic fibrosis [50]. Low retinol levels in NAFLD patients’ liver have been found to be significantly associated with IR [51]. Liver injury and IR in MASH could contribute to the increased Akr1b10, which encodes the key enzyme (aldo‐keto reductase family 1 member B10) of the retinol metabolism [49]. Further, the upregulation of Akr1b10 plays a critical role in MASH development and progression through promoting lipogenesis and eliminating cytotoxic carbonyls [49].

The persistent abnormal expression of cytokines in liver would be activated inflammatory cells to release free radicals, such as reactive oxygen species (ROS) and nitric oxide (NO) reactive species, which can subsequently induce DNA damage, thus fostering neoplastic transformation [52, 53]. In our sequencing data, the upregulated genes *Lpl*, *Cbr3*, *Angpt4*, *Acsl4*, *Srd5a1*, and *Apoa5* and the downregulated genes *Cyp4a10*, *Rdh16*, *Acot1*, *Acot3*, and *Cxcr3* were found to be associated with malignant MASH (Figure 4). As reported, the upregulation of *Lpl*, in cooperation with miR‐467b, affects MASH progression through IR [36]. Lower or absent expression of *Apoa5* was involved in fructose‐induced metabolic dysregulation and was associated with hepatic steatosis [54]. As the rate‐limiting enzyme for bile acid synthesis, CYP7A1 feedback regulates the nuclear hormone orphan receptor farnesoid X receptor (FXR), resulting in the dysregulation of bile acid metabolism that contributes to MASH [55]. Additionally, the high expression levels of *Alox5*, *Pla2g2e*, *Akr1c18*, *Sult1e1, Rdh11, Lpl*, and *Klk1b4* were validated by RT‐qPCR (Figures 5(a), 5(b), 5(c), 5(d)) and were inferred to contribute to adverse MASH progression via the release of inflammatory cytokines (Figure 2(i), Supporting Figure 1D–1I). *Klk1b4, Alox5*, and *Pla2g2e* may interact with cytokines to mediate immune dysfunction, as indicated by PPI network (Figure 5). Further, our results showed upregulation of pro‐inflammatory cytokines IL6, Ccl2, Cxcl1, and Ccl11 and downregulation of anti‐inflammatory cytokines IL2/3/10/12p70/13 and Csf2 (Figure 2). In our study, the HRAS‐Ngf‐Klk1b4 and HRAS‐Gpx1‐Alox5‐Pla2g2e axes may play possible roles in mediating the promoting effects of HRAS overexpression on the development of MASH (Supporting Figure 2D). Moreover, the *Klk1b4, Alox5*, and *Pla2g2e* genes were identified for the first time as potential biomarkers associated with adverse progression of MASH through their involvement in the release of inflammatory cytokines IL6, Ccl2, and Ccl11 (Figure 5). The clinical database (KM‐Plotter) revealed that high expression of *Alox5* and *Pla2g2e* was significantly associated with poor overall survival in patients with nonalcoholic, non‐hepatitis‐related HCC (Supporting Figure 2E–2F). This finding is consistent with our experimental data and supports the hypothesis that these phospholipid‐modifying enzymes may contribute to the aggressive phenotype of MASH‐HCC. The authenticity and reliability of our conclusions were supported by the evidences that the elevated serum IL6 and Ccl11 in MASH patients have been implicated in IR, steatosis, and liver injury, while Ccl2‐mediated hepatic deletion of Wilms’ tumor 1‐associating protein (WTAP) induced MASH [56–59]. Meanwhile, Alox5 and Pla2g2e have been reported to be involved in lipid oxidation, dysregulated lipid metabolism, and inflammation processes, but they have not been reported to be with MASH [60, 61]. Alox5 is expressed in leukocytes and catalyzes the formation of leukotrienes (LTs), which are pro‐inflammatory lipid mediators. Phosphorylation at the Ser271 and Ser663 residues was found to promote ALOX5 nuclear translocation, thereby facilitating the production of LTs and contributing to inflammation responses, including the activation of nuclear factor (NF)‐κB and IL6 secretion by adipose tissue [62]. Moreover, as a member of the secreted phospholipase A2s (sPLA2s) family of encoding genes, Pla2g2e acts on phosphatidylethanolamine (PE) and phosphatidylserine (PS), leading to alterations in lipid composition within lipoprotein particles, which eventually moderately facilitates fat deposition in tissues [61]. The concordance between our preclinical data and these human survival outcomes strengthens the potential of *Alox5* and *Pla2g2e* as both prognostic biomarkers and therapeutic targets in the MASH‐HCC subtype.

In addition, the *Klk1b4* gene, also known as NGFα, encodes the alpha subunit of the 7S nerve growth factor (NGF) complex, whose biological function remains unknown. It is also unable to retrieve reliable prognostic data for *Klk1b4* from the publicly available HCC datasets. This limitation may be attributed to several factors. *Klk1b4* is a member of the tissue kallikrein family, and its expression profile in human liver cancer tissues may be insufficiently captured by the microarray platforms included in these databases. It is possible that *Klk1b4* exhibits low or variable expression in human HCC compared to the rodent model used in our initial discovery, reflecting potential species‐specific differences in lipid metabolism or inflammatory pathways. Alternatively, its functional role may be more prominent in early disease stages not adequately represented in survival datasets. Future studies utilizing RNA sequencing data from larger, well‐annotated MASH‐HCC cohorts or experimental validation in human liver organoids and clinical specimens will be necessary to clarify the role of *Klk1b4* in human. Overall, our work revealed that *Klk1b4*, *Alox5*, and *Pla2g2e* have potential value as candidate biomarkers for MASH progression and warrant further investigation.

The common limitation of MASH research was lack of clinical patients’ sample. Early‐stage samples of MASH are difficult to obtain due to the high likelihood of underdiagnosis or misdiagnosis [20]. Therefore, the findings in our study provide valuable reference for further investigations. Simultaneously, additional samples and experimental validations are necessary to enable more comprehensive and in‐depth analysis. Next, we will examine the expression of *Klk1b4, Alox5*, and *Pla2g2e* in the blood of HRAS mouse model and seek suitable clinical blood samples for validation to further confirm their application value.

In all, we used a novel murine model of MASH tends to HCC with 100% incidence to elucidate the detailed holistic molecular signature. Furthermore, we proposed *Klk1b4, Alox5*, and *Pla2g2e* as novel potential biomarkers of MASH prone to malignancy firstly, while hypothesized that *Klk1b4, Alox5*, and *Pla2g2e* may facilitate the release of IL6, Ccl2 and Ccl11, thereby contributing to the development of malignant MASH and offering potential value in diagnosis and prognostic evaluation.

## 4. Materials and Methods

### 4.1. Mice

The HRAS knock‐in mice (*n* = 58, 5 weeks old) were supplied by the Institute for Laboratory Animal Resources of National Institutes for Food and Drug Control (NIFDC, CHINA). TAM was used to induce liver‐specific *HRAS* gene expressed in HRAS knock‐in mouse and corn oil as solvent at 5 weeks old. HRAS overexpression induced by TAM group is named HRAS^TAM^ group (*n* = 37) for malignant MASH, and that by corn oil group is named HRAS^non−TAM^ group (*n* = 20) as control. All animals were allowed free access to water and diet and provided with a 12‐h light/dark cycle. All animal experiments were approved by the Experimental Animal Committee of NIFDC (approval number: 2022‐B‐050).

### 4.2. Serum Biochemical Index Detection

Blood was sampled from HRAS^TAM^ (*n* = 6) and HRAS^non−TAM^ (*n* = 8) mice allowed to fast for 4 h, and serum was separated by centrifugation. The biochemical index was detected by the automatic blood and biochemistry analysis instrument (OLYMPUS AU400): HRAS^TAM^ (*n* = 6) and HRAS^non−TAM^ (*n* = 8).

### 4.3. ITT

Fast mice for at 3 h before insulin injection, while ensuring that the mice have access to drinking water. Dilute insulin in 0.9% NaCl and prepare 20% glucose (D‐(+)‐glucose solution dissolved in distilled water) to be administered if the mice become hypoglycemic. Measure blood glucose levels at selected time points after inject insulin intraperitoneally (0.75 U insulin/kg body weight; after 0, 15, 30, 45, 60, 75, 90, 105, and 120 min).

### 4.4. Real‐Time Quantitative PCR

To evaluate the expression level of a gene by RT‐qPCR, total RNA was extracted from mouse tissues by RNAisoPlus. RNA concentration was measured by NanoDrop. Then, cDNA was converted by PrimeScript RT reagent kit with gDNA Eraser Kit. Real‐time PCR was performed on Roche quantitative PCR instrument with TB Green Premix Ex Taq (Takara, RR047B). Glyceraldehyde‐3‐phosphate dehydrogenase (GAPDH) was used as the endogenous control. The primers used to determine mRNA in tissues of mice are listed in Supporting Table 1. Mean levels of Cq values were measured from triplicate PCR analyses for each sample, and relative quantification method was used for RT‐qPCR data analysis. Each sample was repeated three times (*n* = 3).

### 4.5. Protein Extraction and Western Blot Analysis

To extract total proteins, tissues were lysed in tissue total protein lysis buffer kit (Sangon, C500028). The BCA Protein Assay Kit was used to detect the protein concentration in tissues of HRAS^TAM^, HRAS^non−TAM^ mice, and C57BL/6 mice. Protein solutions were boiled in 5 × loading buffer at 98 °C for 10 min and then resolved by SDS‐PAGE. All the primary antibodies were diluted in 3% BSA; the dilution ratio was followed by: HRAS‐specific polyclonal antibody (1:1,000, Proteintech) and GAPDH antibody (1:10,000, Abcam). Primary antibodies were detected by goat anti‐rabbit IgG‐HRP‐conjugated secondary antibody (Santa Cruze,1:10,000). Chemiluminescence images were captured using glue applicator. Each sample was repeated three times (*n* = 3).

### 4.6. Inflammatory Cytokines Detection

The blood samples collected at week 1 (after modeling 7 days) of HRAS^TAM^ mice (*n* = 6) and HRAS^non−TAM^ mice (*n* = 4) were used to separate serum at 10,000 rpm. The serum was used for the determination of cytokines using a Bio‐Plex 200 system and Bio‐Plex Pro Mouse Cytokine 23‐plex Assay (M60009RDPD, Bio‐Rad, CA, USA), according to the instruction manual (10014905, Bio‐Rad, CA, USA).

### 4.7. Hematoxylin‐Eosin Staining (H&E)

The liver tissues of HRAS^TAM^ mice (*n* = 3) and HRAS^non−TAM^ mice (*n* = 3) at week 1 were fixed in formalin, embedded in paraffin, and transferred to 70% ethanol. Individual lobes of liver tissue biopsy material were placed in processing cassettes, dehydrated through a serial alcohol gradient, and embedded in paraffin wax blocks. Before immunostaining, 5‐μm‐thick liver tissue sections were dewaxed in xylene, rehydrated through decreasing concentrations of ethanol, washed in PBS, and then stained with H&E staining kit (Leagene, DH0003). The sections were scanned by NanoZoomer (HAMAMATSU; C13210‐01) and previewed by Nano Zoomer Digital Pathology software (NDP.viewer).

### 4.8. Lipid Staining

The mice liver samples were treated using 4% paraformaldehyde for frozen sectioning (−15°C). The 60% isopropanol immersion was performed on 8‐μm sections. Seal dyeing with Oil Red O dye solution for 10 min (Leagene, DL0011). The 60% isopropanol was washed and counterstained with hematoxylin. The histopathological examination of liver was performed under an Olympus BH2 microscope. Bodipy 493/503 (1 mg/mL, D3922, Thermo Fisher Scientific) was used for staining of neutral lipids in tissue sections. The sections were scanned by NanoZoomer (HAMAMATSU; C13210‐01) and previewed by NDP.viewer.

### 4.9. Immunohistochemistry, IHC

The most typical mice of HRAS^TAM^ and HRAS^non−TAM^ groups per week (at least 2 mice) were selected to detect the expression of markers. For immunohistochemistry, liver tissue paraffin section was dewaxed through xylene and a serial alcohol gradient and incubated in 3% H_2_O_2_ for 15 min, boiled with citrate buffer (pH 6.0) for antigen retrieval. Then, after blocking with fetal bovine serum, the following primary antibodies were incubated overnight at 4 °C: HRAS‐specific polyclonal antibody (1:100, Proteintech); lipoprotein lipase antibody (LPL, 1:500, Genetex). After PBS washing and incubating the secondary antibodies for 1 h at 37°C, it was sealed with glycerin. The sections were scanned by NanoZoomer (HAMAMATSU; C13210‐01) and previewed by NDP.viewer.

### 4.10. RNA Sequencing

RNA sequencing was performed on two groups by HRAS^TAM^ (*n* = 5) and HRAS^non−TAM^ (*n* = 4). The samples were sequenced by the Illumina NovaSeq 6000. All the downstream analyses were based on the clean data with high quality. Differential expression analysis of two groups was performed using the DESeq2 R package (1.20.0). The *p* values were adjusted using the Benjamini & Hochberg method. Padj ≤ 0.05 and |log2(foldchange)| ≥ 1.5 were set as the threshold for significantly differential expression. ClusterProfiler R package (3.8.1) was used to test the statistical enrichment of differentially expressed genes in KEGG pathways, with corrected *p* value less than 0.05 considered significantly enriched by differentially expressed genes. Gene Ontology (GO) enrichment analysis of differentially expressed genes was implemented by the cluster Profiler R package (3.8.1), in which gene length bias was corrected. GO terms with corrected *p* value less than 0.05 were considered significantly enriched by differentially expressed genes. Shared DEGs were imported into the STRING (Version 10.0, https://www.string-db.org/) web tool to map the protein–protein interaction (PPI) network. RNA sequencing was co‐operated with Novogene Co., Ltd.

### 4.11. Statistical Analysis

All statistical analyses were performed using Prism V.8.0 (GraphPad, La Jolla, California, USA). All data are shown as mean ± standard deviation (SD). Statistical significance between two groups was calculated using unpaired, Student *t* test. ^∗^ *p* < 0.05 was considered statistically significant (^∗^ *p* < 0.05; ^∗∗^ *p* < 0.01; ^∗∗∗^ *p* < 0.001; ^∗∗∗∗^ *p* < 0.0001; ^ns^: no statistical difference). Most experiments were carried out at least three times, and the findings of all key experiments were reliably reproduced.

## Author Contributions

Conceptualization, Changfa Fan and Yuwei Zhao; writing–original draft, Changfa Fan, Chen Ling, and Yuya Wang; all experiments, Chen Ling; mouse modeling experiments, Susu Liu and Haoyang Zhao; functional experiments, Nan Xu and Hong Wang; pathological diagnosis, Guitao Huo, and Yanwei Yang; statistical analysis, Zeqiang Zhang; writing–review and editing, Yuwei Zhao; resources, Rui Fu; supervision, Yuwei Zhao and Changfa Fan.

## Funding

This work was supported by National Institutes for Food and Drug Control, State Key Laboratory of Drug Regulatory Science, Grant/Award Numbers: GJJSYJ202401 and 2023SKLDRS0124.

## Ethics Statement

The animal studies were approved by NIFDC in China. All animal experiments were approved by the Experimental Animal Committee of NIFDC (approval number: 2022‐B‐050; 2021‐B‐003). Written informed consent was obtained from the owners for the participation of their animals in this study.

## Conflicts of Interest

The authors declare no conflicts of interest.

## Supporting Information

Additional supporting information can be found online in the Supporting Information section.

## Supporting information


**Supporting Information** Supporting table 1. The primer sequences for mRNA detection in RT‐qPCR analysis. This supporting information provided the primer sequences used for amplification of target genes, which are essential for mRNA detection in RT‐qPCR analysis. Supporting table 2. The quality control of RNASeq. This supporting information provided quality control information for the RNA‐Seq data, ensuring the reliability and scientific validity of the sequencing results. Supporting table 3. The information of DEGs and the inserted human HRAS gene of RNASeq. This supporting information provided information for DEGs and the inserted human HRAS gene of RNA‐Seq data, ensuring the reliability and scientific validity of the sequencing results. This supporting information provides essential data that support the results shown in Figure 4A–B. Supporting table 4. The information of KEGG and GO analysis of RNASeq. This supporting information provided information for KEGG and GO analysis of RNA‐Seq data, ensuring the reliability and scientific validity of the sequencing results. This supporting information provides essential data that support the results shown in Figure 4C–4F.

## Data Availability

The raw sequencing data reported in this paper are deposited in NCBI (PRJNA1098413), Chinese National Center for Bioinformation (PRJCA025073), and Science Data Bank (DOI: 10.57760/sciencedb.j00139.00132).
