# Supplementary material for: Novel Potential Markers of Metabolic Dysfunction–Associated Steatohepatitis Prone to Hepatocellular Carcinoma
Source: Can J Gastroenterol Hepatol. 2026 May 26;2026:7390933. doi: 10.1155/cjgh/7390933 (PMC13203792; doi:10.1155/cjgh/7390933)
Supplement: Supplementary file 1 — Supporting Information Supporting table 1. The primer sequences for mRNA detection in RT‐qPCR analysis. This supporting information provided the primer sequences used for amplification of target genes, which are essential for mRNA detection in RT‐qPCR analysis. Supporting table 2. The quality control of RNASeq. This supporting information provided quality control information for the RNA‐Seq data, ensuring the reliability and scientific validity of the sequencing results. Supporting table 3. The information of DEGs and the inserted human HRAS gene of RNASeq. This supporting information provided information for DEGs and the inserted human HRAS gene of RNA‐Seq data, ensuring the reliability and scientific validity of the sequencing results. This supporting information provides essential data that support the results shown in Figure 4A–B. Supporting table 4. The information of KEGG and GO analysis of RNASeq. This supporting information provided information for KEGG and GO analysis of RNA‐Seq data, ensuring the reliability and scientific validity of the sequencing results. This supporting information provides essential data that support the results shown in Figure 4C–4F. [file CJGH-2026-7390933-s001.zip › supplyment table 1.docx]

Supplementary Table 1. The primer sequences for mRNA detection in RT-qPCR analysis.

| Oligonucleotides | Forward primer | Reversed primer |
| --- | --- | --- |
| *Lpl* | GGGAGTTTGGCTCCAGAGTTT | TGTGTCTTCAGGGGTCCTTAG |
| *Apoa5* | TCCTCGCAGTGTTCGCAAG | CGAAGCTGCCTTTCAGGTTCT |
| *Cyp7a1* | GGGATTGCTGTGGTAGTGAGC | GGTATGGAATCAACCCGTTGTC |
| *Pla2g2e* | CCAGTGGACGAGACGGATTG | GTTGTGGCGAAAGCAGAGAG |
| *Akr1c18* | TCGTCCAGAGTTGGTCAGAC | CTTTAGGCAAAAGCTCATTCCCT |
| *Alox5* | ACTACATCTACCTCAGCCTCATT | GGTGACATCGTAGGAGTCCAC |
| *Sult1e1* | ATGGAGACTTCTATGCCTGAGT | ACACAACTTCACTAATCCAGGTG |
| *Rdh11* | ACCAAGTCTATTCGAGCCTTTG | TGACGCCAATGTGCATCTCAA |
| *Gapdh* | CAGCAACTCCCACTCTTCCAC | TGGTCCAGGGTTTCTTACTC |
